# Associations between Maternal Diet, Human Milk Macronutrients, and Breast-Fed Infant Growth during the First Month of Life in the SMILE Iwamizawa in Japan

**DOI:** 10.3390/nu15030654

**Published:** 2023-01-28

**Authors:** Yosuke Komatsu, Yasuaki Wada, Fuka Tabata, Satomi Kawakami, Yasuhiro Takeda, Kiminori Nakamura, Tokiyoshi Ayabe, Koshi Nakamura, Takashi Kimura, Akiko Tamakoshi

**Affiliations:** 1 Health Care & Nutritional Science Institute, Morinaga Milk Industry Co., Ltd., Zama 252-8583, Japan; 2 Center for Food and Medical Innovation Promotion, Institute for the Promotion of Business-Regional Collaboration of Hokkaido University, Sapporo 001-0021, Japan; 3 Department of Cell Biological Science, Faculty of Advanced Life Science, Department of Cell Biological Science, Hokkaido University Graduate School of Life Science, Sapporo 001-0021, Japan; 4 Department of Public Health and Hygiene, Graduate School of Medicine, University of the Ryukyus, Okinawa 903-0215, Japan; 5 Department of Public Health, Hokkaido University Graduate School of Medicine, Sapporo 060-8638, Japan

**Keywords:** human milk, maternal diet, macronutrient, infant growth, breast-fed infant

## Abstract

Maternal diet may affect human milk macronutrients, but it remains to be elucidated whether this is also influential in infant growth. This study aimed to examine (1) how maternal diet influences human milk macronutrients, and (2) to what extent the variation in milk macronutrients affects infant growth during the first month of life. In 71 Japanese lactating women, maternal dietary information was collected from the brief-type self-administered diet history questionnaire, and anthropometry of mother–infant dyads was collected from medical records. Macronutrients in milk were analyzed by a Human Milk Analyzer. Maternal retinol intake was associated with the carbohydrate content in human milk at 1-month postpartum (standardized *β* coefficient: 0.287; *p* = 0.038). Moreover, the energy content in human milk was associated with an increase in the weight standard deviation score based on the WHO growth standard at 1 month of age (standardized *β* coefficient: 0.399; *p* = 0.046). Nevertheless, the milk macronutrient was not associated with the risk of infant growth abnormalities. In conclusion, a part of the maternal diet impacts macronutrient contents in human milk, but milk macronutrients have a limited effect on infant growth only within the normal growth curve during the first month of life.

## 1. Introduction

There has been a worldwide consensus authorized by the World Health Organization (WHO) that breastfeeding is the optimal mode of infant feeding during the early period of life [1]. It provides the infant with a variety of nutrients as well as bioactives that support optimal growth and development [2,3,4]. Breastfeeding during the first 6 months of life is particularly recommended, as it is associated with a reduction in the risks of various kinds of diseases early in life [1,5]. Continued breastfeeding from 6 to 24 months of life, with the use of complimentary food, has also been encouraged, as this also contributes to the reduced risks of infectious diseases early in life and non-communicable diseases later in life [1]. Whereas breastfeeding is the gold standard of infant feeding, infant formulas (IFs) are commonly used in case the availability of the mother’s own milk is limited. The majority of IF products are formulated using cow milk proteins because of its high nutritional value. With an emphasis on making the growth and development patterns of formula-fed infants closer to those of breast-fed infants, their compositions have been revised continuously in the past decades with reference to increasing evidence of infant nutrition [6,7,8].

The period of 1000 days from conception to two years of age is important for the healthy growth and development of infants/children [9]. Growth patterns are different between breast-fed and formula-fed infants [10,11,12], despite years of compositional updates of IFs, as described above. Specifically, formula-fed infants gain more weight for length compared with breast-fed infants in the first year of life, which is attributed to the larger amount of lean mass in the former compared with the latter [12]. Although fat mass in formula-fed infants was lower at 3–4 and 6 months than in breast-fed infants, at 12 month, fat mass in formula-fed infants was higher than in breast-fed infants [12], which would have also contributed to the increase in weight for length in formula-fed infants in the first year of life. Growth trajectories could also be different even between breast-fed infants/children, depending on the quantity and/or quality of human milk provided to them. The issue of human milk quantity mostly occurs for exclusively breast-fed neonates; breastfeeding insufficiency is often observed following maternal incapability of copious milk production, which increases the risk of severe neonatal weight loss and early readmission as a result [13]. Mainly, it is known that advanced maternal age and excessive weight gain during pregnancy could lead to delayed milk production, increasing the risk of neonatal weight loss [14,15]. What initially needs to be kept in mind about human milk quality is that it is a dynamic fluid, the composition of which dramatically changes during the lactation period [16]. Moreover, human milk compositions are influenced by other maternal factors, such as age, ethnicity, pre-pregnancy anthropometry, mode of delivery, and diet (including time elapsed since last meal) [17,18]. However, investigations are quite limited on how compositional variances of human milk would affect the growth and development of infants/children. Gridneva et al. reported that carbohydrate concentration in human milk was positively associated with infant length, weight, and fat-free mass and negatively with fat mass during the first year of life, while total carbohydrate intake was positively associated with fat mass [19]. Another study by Young et al. found that protein concentration in human milk was inversely associated with fat mass until 4 months of age of term-born infants [20]. A recent study by de Fluiter et al. observed that higher fat and energy content was associated with a higher gain in fat from age 1 to 6 months in term-born infants [21]. Still, data on human milk macronutrients in relation to anthropometry of infants/children are scarce, requiring further investigation.

As a small part of SMILE Iwamizawa (Survey on Mothers, Infants, and Children Lives and Environments in Iwamizawa, Hokkaido, Japan), in the present study, we aimed to examine (1) how maternal diet influences the macronutrients of human milk, and (2) to what extent the variation in human milk macronutrients affects infant growth at 1 month after birth. Maternal dietary information was collected from the brief-type self-administered diet history questionnaire (BDHQ) [22], macronutrient compositions of human milk were analyzed by a Human Milk Analyzer, and anthropometry of mother–infant dyads was collected from medical records.

## 2. Materials and Methods

### 2.1. Study Design and Participants

The present study was a performed as a small part of the SMILE Iwamizawa, a cohort study in process in Iwamizawa in Hokkaido, Japan. This cohort study aims to examine healthy mother–infant/child dyads during pregnancy and postpartum in order to clarify the environmental factors that influence infants’ growth and development. Pregnant women living Iwamizawa were recruited at the time when the municipal government of Iwamizawa issued the Mother and Child Health Handbooks to women. This study was conducted according to the guidelines laid down in the Declaration of Helsinki and all procedures involving human subjects were approved by the ethics committees of the Graduate School of Medicine, Hokkaido University, and the Morinaga Milk Industry (approval numbers 16-039 and 16005-144, respectively). Written informed consent was obtained from all subjects. Infants’ research consents were deemed based on consent signatures by their mothers.

**Figure 1:**
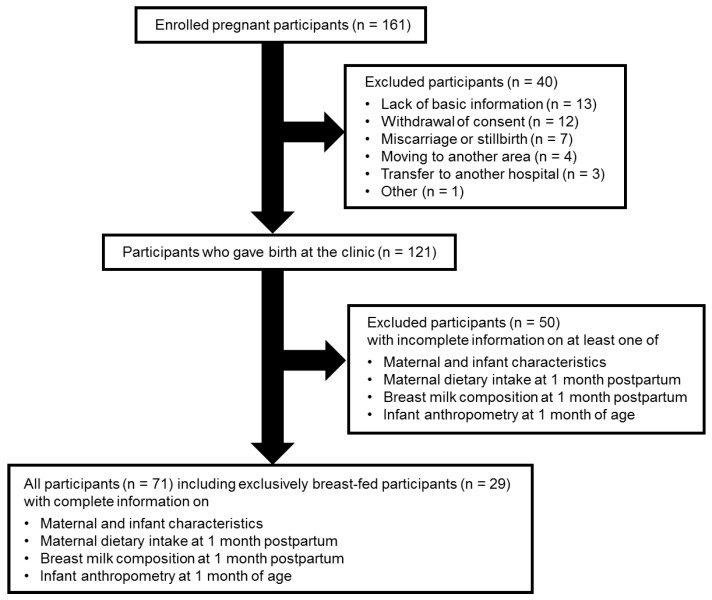
Flow chart of this study diagram.

A total of 161 pregnant women who visited the Iwamizawa Ladies Clinic between June 2017 and January 2020 agreed to participate in the study with written informed consent obtained; 121 participants gave birth at the clinic; 40 participants were excluded due to lack of basic information (n = 13), withdrawal of consent (n = 12), miscarriage or stillbirth (n = 7), moving to another area (n = 4), transfer to another hospital (n = 3), or other reasons (n = 1). Furthermore, 50 participants with incomplete information on at least one of maternal and infant characteristics, maternal dietary intakes at 1-month postpartum, human milk macronutrients at 1-month postpartum, and infant anthropometry at 1 month of age were excluded from subsequent analyses (Figure 1). Infants’ research consent was inferred based on consent signatures by their mothers. Maternal dietary information, anthropometry data, and human milk were collected as described later. All 71 participants had complete information on maternal and infant characteristics, maternal dietary intakes at 1-month postpartum, human milk macronutrients at 1-month postpartum, and infant anthropometry at 1 month of age, and 29 of these infants were exclusively breast-fed during the month. In the questionnaire at the 1-month checkup, 29 participants who responded that they had been exclusively breastfeeding during the first month were selected as exclusively breast-fed mother–infant dyads.

### 2.2. Dietary Assessment

The BDHQ was used for the dietary survey on the participants at 1-month checkup. The BDHQ is a questionnaire designed to obtain information on the amount of nutrients habitually consumed in the diet of people living in Japan over the past month [23]. The validation of the BDHQ was performed by using the 16-day weighed dietary record method, in which meals were weighed and recorded for a total of 16 days, 4 days in each season throughout the year [23]. Energy adjustment was performed using the residual method, which is calculated using linear regression with total energy intake as the independent variable and the intakes of food and nutrients as the dependent variable [24].

### 2.3. Human Milk Collection and Analysis

Human milk samples were collected during the hospitalization period immediately after delivery (viewed as “colostrum” in the present study), and at 1-month postpartum. Human milk samples were collected several times (up to 5 times) at timings around 1-month postpartum. The mean values of macronutrients in these samples were used in the subsequent analysis in order to consider the effects of collection timing, such as time elapsed since last meal and daily variation. Human milk was collected by each participant by using a breast pump (Pigeon, Tokyo, Japan) according to the manufacturer’s instructions; the samples were transferred to a 50 mL tube and temporarily stored in a freezer at home or the clinic, and then transferred to the laboratory and stored at −80 °C until analysis. Human milk analysis was conducted with reference to our previous study [25]. All milk samples were thawed, homogenized using the ultrasonic MIRIS sonicator (MIRIS AB, Uppsala, Sweden), and maintained at 40 °C prior to analysis. The concentrations of energy, protein, fat, and carbohydrate in the homogenized human milk samples (3 mL) were measured using the Human Milk Analyzer (MIRIS AB) with a medium infrared transmission spectroscopy technique.

### 2.4. Anthropometry Data

Anthropometry data of mother–infant dyads were obtained from their medical records. For infants, the length, head circumference, and chest circumference were manually measured with a measure, and the weight was measured with an automatic scale (MY-230, Kubota, Osaka, Japan) at birth and the 1-month checkup at Iwamizawa Ladies Clinic, respectively.

### 2.5. Statistical Analyses

JMP software (version Pro 14.0.0, SAS Institute, Cary, NC, USA) was used for statistical analyses. Continuous variables are presented as the mean ± standard deviation (SD), and Student t-tests with Welch’s correction were used to compare the means of each group. Categorical variables are shown as the number and percentage, and the χ^2^ test was used to compare the data in percentage between groups. The SD score (SDS), calculated with the mean as 0 and the SD as 1, was used as an index of anthropometry data of infants. The SDSs of length and weight based on Japanese growth standards were calculated using a Microsoft Excel-based tool for growth evaluation provided by the Japanese Society for Pediatric Endocrinology [26], and reference data at 1 month of age were used for infants aged 25–41 days. The SDSs of length, weight, weight for length (an objective indicator of an infant’s physical growth), and head circumference based on WHO growth standards were calculated using Peditool, a web-available calculation tool for growth evaluation adjusted for gestational age [27]. For length, weight, weight for length, and head circumference, the calculated SDSs were used for analyses in addition to the measured values. For chest circumference, the SDS was not calculated due to the lack of Japanese and WHO growth standards; therefore, only measured values were used for analysis. Linear regression analyses were performed to examine the association between maternal dietary intakes and human milk macronutrients as well as the association between human milk macronutrients and infant growth. The model incorporated the following covariates as potential confounding factors: maternal age (years), early-pregnancy body mass index (BMI) (kg/m^2^), gestational weeks, parity (primipara or not), mode of delivery (natural or caesarean), maternal weight gain (kg), infant sex (male or female), and infant birth weight (g). Logistic regression analyses were performed to examine human milk macronutrients for the risk of infant growth abnormalities (defined here as out of the 10th–90th percentile in length and weight based on the Japanese or WHO growth standards), with the model incorporating the same covariates used in the earlier analyses. All probability values were two-tailed, and *p* values of <0.05 were considered statistically significant.

## 3. Results

### 3.1. Participant Characteristics and Maternal Dietary Intakes

Maternal and infant characteristics of the participants with complete information on maternal and infant characteristics, maternal dietary intakes, human milk macronutrients, and infant anthropometry are summarized in Table 1 (n = 71). Maternal food intake and nutrient intake at 1-month postpartum, after adjusting for the energy intake by the residual method, are shown in Supplementary Tables S1 and S2, respectively (n = 71). The mean ± SD of energy intake reported at BDHQ was 1594.5 ± 448.7 kcal/day, and energy-adjusted protein, fat, and carbohydrate intakes were 58.4 ± 8.3 g/day, 50.5 ± 7.7 g/day, and 220.3 ± 24.2 g/day, respectively.

### 3.2. Macronutrients in Human Milk

Macronutrient compositions in colostrum and human milk at 1-month postpartum are shown in Table 2 (n = 71). The energy content, fat, and carbohydrate concentrations in human milk at 1-month postpartum were significantly higher than in colostrum. In contrast, the protein concentration in human milk at 1-month postpartum was significantly lower than in colostrum.

### 3.3. Association between Maternal Dietary Intakes and Human Milk Macronutrients

The associations between maternal food intake and nutrient intake at 1-month postpartum and human milk macronutrients at 1-month postpartum were examined using multivariate linear regression analyses (Supplementary Table S3 and Table 3, respectively). The intake of raw fish was negatively and significantly correlated with the energy content and fat concentration in human milk (standardized *β* coefficient: −0.287, *p* = 0.028 and standardized *β*: −0.280, *p* = 0.032, respectively). In addition, the retinol intake and retinol activity equivalent (RAE) intake were positively and significantly correlated with carbohydrate concentration in human milk (standardized *β*: 0.287, *p* = 0.038 and standardized *β*: 0.306, *p* = 0.022, respectively).

### 3.4. Gestational Outcome and Anthropometry of Infants

Gestational outcome and anthropometry of the breast-fed (n = 29) and mixed-fed (n = 42) infants are shown in Table S4. There were no significant differences in gestational outcome between the two groups. Furthermore, measured values and SDSs of anthropometry such as length, weight, head circumference, and chest circumference at 1 month of age did not show significant differences between the two groups. Similarly, changes in measured values and SDSs of anthropometry also did not show significant differences between the two groups.

### 3.5. Association between Human Milk Macronutrients and Breast-Fed Infant Growth

The association between human milk macronutrients at 1-month postpartum and breast-fed infant growth at 1 month of age was examined using multivariate linear regression analysis (Table 4). The exclusively breast-fed mother–infant dyads, with complete information on maternal and infant characteristics, maternal dietary intakes, human milk macronutrients, and infant anthropometry, were included for this analysis (n = 29) as subgroup analyses. The energy content in human milk was positively and significantly correlated with an increase in the weight SDS based on both the Japanese and WHO growth standards at 1 month of age (standardized *β*: 0.326, *p* = 0.044 and standardized *β*: 0.399, *p* = 0.046, respectively).

Moreover, the carbohydrate concentration in human milk was positively and significantly correlated with an increase in chest circumference at 1 month of age (standardized *β*: 0.540, *p* = 0.044).

### 3.6. Association between Human Milk Macronutrients and the Risk of Infant Growth Abnormalities

The association between human milk macronutrients at 1-month postpartum and the risk of breast-fed infant growth abnormalities at 1 month of age was examined (Table 5). The exclusively breast-fed mother–infant dyads, with complete information on maternal and infant characteristics, maternal dietary intakes, human milk macronutrients, and infant anthropometry, were included for the analysis (n = 29). Logistic regression models indicated that there was no correlation between human milk macronutrients and the risk of infant growth abnormalities.

## 4. Discussion

Nutritional compositions in human milk are known to be influenced by many factors, but it remains to be elucidated how the variation in macronutrients in human milk would affect the growth and development of exclusively breast-fed infants. With an emphasis on the fact that the macronutrient composition of human milk can fluctuate under the influence of maternal diet [28], we initially examined the associations between maternal dietary intakes and human milk macronutrients. We also investigated whether or not differences in human milk macronutrients would influence the growth of breast-fed infants during the first month of life.

The maternal energy intake (1594.5 kcal/day) obtained by BDHQ in this study was lower than that (1799 kcal/day) of lactating women in the National Health and Nutrition Survey in Japan [29]. Although the BDHQ method has been fully validated [23], it is known to estimate lower absolute values than that estimated by the dietary record method used in the National Health and Nutrition Survey [30]. Adjustment was made for food and nutrient intakes by energy intake through the residual method in the present study. The macronutrient compositions of colostrum and human milk at 1-month postpartum in the present study were comparable to those reported in other cohort studies [2,31,32,33,34,35,36,37]. The energy content of human milk at 1-month postpartum was higher than that of colostrum, whereas the protein concentration was lower at 1-month postpartum than that of colostrum. These observations are in line with previous reports [2,34,35,36]. The milk fat content is the most variable of the macronutrients and increases during the first month after birth [32,34], and this trend was also observed in our study. When it comes to carbohydrates, previous studies have reported a decrease in the oligosaccharide content and an increase in the lactose content in human milk, resulting in an increase in overall carbohydrates during the first month after birth [32,37]. Although the composition of carbohydrates, such as oligosaccharides and lactose, was not analyzed here, the overall carbohydrate content of human milk also increased during the first month in our study. Taken together, variations in human milk macronutrients are consistent with previous surveys and reports, supporting the validity of this study.

The maternal raw fish intake was negatively and significantly correlated with the energy content and fat content in human milk. The habitual consumption of raw fish in Japan has been declining in recent years [38], possibly resulting in an increase in the variation of its intake. Although mechanisms are not clear that link maternal fish consumption and energy and fat content in human milk, these associations might be manifested here as a result of increased variance of fish consumption among the Japanese population. It should be noted that several studies on the association between maternal diet and human milk components have already been reported [39,40]. They focused on the association between the Italian Mediterranean diet and lipid components such as n-3 fatty acids in human milk. Although the lipid fractions in human milk were not analyzed in the present study, the Japanese maternal diet we investigated might also have influenced such lipid components. The maternal retinol intake and RAE intake were positively and significantly correlated with the carbohydrate content in human milk. However, none of the foods were significantly correlated with the carbohydrate content in human milk. This implies that the effect of retinol intake may not have been derived from a specific food, but from a combination of several foods. It should be emphasized that the mechanisms by which retinol intake affects carbohydrate levels in human milk remain unknown. A previous observational study in lactating women reported a positive correlation between the intake of vitamin A, including retinol, and the sialic acid level in human milk [41]. Sialic acid is known to be involved in the synthesis of the oligosaccharide sialyllactose [42,43], which is a part of carbohydrates. However, since the principal carbohydrate in human milk is lactose (7 g/100 mL) [4] and the content of sialyllactose is quite smaller (13.5–215 mg/100 mL) [43], the contribution of only sialyllactose to the increase in the carbohydrate content in milk is likely to be very limited. Similar associations between retinol and other oligosaccharides would be implicated, and further investigation is needed to elucidate them.

There were no significant differences in the anthropometry data and their changes between the breast-fed and mixed-fed infants at 1 month of age in the present study. Formula-fed infants have been reported to have an increased body weight and weight for length compared with breast-fed infants [10,44,45]. Putet et al. reported no significant differences in the weight and length between breast-fed and formula-fed infants at 2 weeks of age in a study on a population of healthy and full-term infants [46]. Thus, the differences in anthropometry between breast-fed and mixed-fed infants in the present study may not have been clear at 1 month of age. The energy content in human milk was positively and significantly correlated with an increase in the weight SDS at 1 month of age. Furthermore, the carbohydrate content in human milk was positively and significantly correlated with an increase in the chest circumference at 1 month of age. These findings suggest that higher energy and carbohydrates in human milk could promote infant growth as early as the first month after birth. Previous reports have shown that weight gain during infancy is associated with an increased risk of obesity in childhood and young adulthood [47,48]. Notably, none of the logistic regression models indicated an association between the macronutrient content in human milk and infant growth abnormalities. Taken together, the energy content and carbohydrate content in human milk were linearly associated with a relative weight gain and an increase in the chest circumference within the normal growth curve during the first month of life, but these were not associated with the risk of growth abnormalities.

A limitation of this study is that the amount of human milk ingested by the infants was not measured, and the associations between human milk composition and infant growth were only analyzed in the subgroup of exclusive breastfeeding. It is essential to measure the amount of human milk intake in order to examine the effect of human milk macronutrients on infant growth in greater detail. Another limitation is that our study participants were recruited at a single clinic in the northern part of Japan. Therefore, there may have been selection bias that may have influenced the results of this study. In fact, the birth size of infants in our study was larger than that in the general population of infants in Japan. The proportions of a low-birth-weight delivery (2.8%) and preterm birth (1.4%) in this study were lower than those previously reported in a Japanese nationwide survey (9.4% [49] and 5.6% [50], respectively). Furthermore, infants born small for gestational age and large for gestational age, defined as the birth weight < 10th percentile and ≥90th percentile for gestational age, were 4.2% and 25.4%, respectively. It is possible that the above bias of infant backgrounds may have influenced the results of this study. Further large-scale, meticulous investigations are warranted to verify the associations we observed in this study.

In conclusion, the maternal retinol intake and RAE intake were associated with carbohydrate content in human milk at 1-month postpartum. Furthermore, the energy content and carbohydrate content of human milk were associated with an increase in the weight SDS and chest circumference at 1 month of age. In contrast, the macronutrient composition in human milk was not associated with the risk of infant growth abnormalities. These findings indicate that part of the diet of lactating women affects the carbohydrate content of human milk, but the energy content and carbohydrate content of the human milk can have a limited effect on infant growth only within the normal growth curve. This study focused only on the first month of life and the first study of the SMILE Iwamizawa. The association between maternal nutrient intakes and the content of macronutrients in human milk, and between these contents and long-term infant/child growth, can be elucidated further through the follow-up of this cohort.

## Figures and Tables

**Table 1:** Maternal and infant characteristics.

Characteristics (n = 71)	
Maternal background	
Age (years) *^1^	31.1 ± 4.9
Height (cm) *^1^	158.6 ± 5.6
Early-pregnancy BMI *^2^ (kg/m^2^) *^1^	21.6 ± 3.2
Weight gain during pregnancy *^3^ (kg) *^1^	10.6 ± 3.0
Gestational period (weeks) *^1^	39.5 ± 1.2
Primipara [n (%)]	28 (39.4)
Caesarean delivery [n (%)]	10 (14.1)
Infant background	
Male [n (%)]	41 (57.7)
Birth weight (g) *^1^	3239.6 ± 391.6
Birth weight for gestational age *^4^	
SGA [n (%)]	3 (4.2)
LGA [n (%)]	18 (25.4)
Low birth weight delivery [<2500 g; n (%)]	2 (2.8)
Preterm delivery [<37 weeks; n (%)]	1 (1.4)

*^1^ Means ± SDs. *^2^ Calculated based on information obtained at the first clinical visit. *^3^ Difference between the weight measured at the first clinical visit and just before delivery. *^4^ SGA and LGA were defined as the birth weight < 10th percentile and ≥ 90th percentile for gestational age, respectively. BMI, body mass index; LGA, large for gestational age; SGA, small for gestational age.

**Table 2:** Macronutrients in colostrum and human milk at 1-month postpartum.

(n = 71)	Colostrum	Human Milk at 1-Month Postpartum	*p*
Energy (kcal/100 mL) *^1^	56.1 ± 17.4	70.5 ± 13.9	<0.001
Protein (g/100 mL) *^1^	1.9 ± 0.9	1.2 ± 0.4	<0.001
Fat (g/100 mL) *^1^	2.2 ± 1.4	3.7 ± 1.5	<0.001
Carbohydrate (g/100 mL) *^1^	6.8 ± 0.9	7.6 ± 0.7	<0.001

*^1^ Means ± SDs.

**Table 3:** Multivariable linear regression analysis for maternal dietary intakes and human milk macronutrients at 1-month postpartum *^1^.

(n = 71)	Human Milk Macronutrient							
	Energy		Protein		Fat		Carbohydrate	
	*β* *^2^	*p*	*β* *^2^	*p*	*β* *^2^	*p*	*β* *^2^	*p*
Dietary nutrient intake								
Protein	0.108	0.429	−0.022	0.873	0.116	0.396	−0.006	0.968
Animal protein	0.070	0.586	−0.038	0.766	0.071	0.582	0.030	0.816
Vegetable protein	0.068	0.626	0.053	0.702	0.085	0.540	−0.097	0.488
Fat	0.199	0.124	0.009	0.943	0.176	0.175	0.099	0.454
Animal fat	0.094	0.467	−0.121	0.350	0.083	0.525	0.137	0.297
Vegetable fat	0.170	0.174	0.138	0.269	0.151	0.229	−0.009	0.943
Saturated fat	0.116	0.398	−0.071	0.602	0.098	0.473	0.136	0.327
Monounsaturated fat	0.243	0.056	0.059	0.645	0.219	0.088	0.060	0.648
Polyunsaturated fat	0.178	0.192	0.077	0.575	0.151	0.271	0.069	0.620
*n*-3 Polyunsaturated fat	0.045	0.739	−0.063	0.639	0.049	0.720	0.033	0.811
*n*-6 Polyunsaturated fat	0.200	0.139	0.120	0.377	0.166	0.224	0.071	0.610
Cholesterol	0.081	0.535	0.019	0.885	0.068	0.604	0.044	0.738
Carbohydrate	−0.155	0.228	−0.017	0.892	−0.139	0.280	−0.054	0.679
Sucrose	−0.056	0.684	0.040	0.771	−0.054	0.698	−0.038	0.788
Total dietary fiber	0.068	0.627	−0.082	0.555	0.074	0.598	0.115	0.419
Soluble dietary fiber	0.077	0.582	−0.110	0.427	0.077	0.580	0.176	0.211
Insoluble dietary fiber	0.073	0.602	−0.069	0.618	0.080	0.569	0.095	0.502
Minerals	0.026	0.845	−0.151	0.250	0.025	0.852	0.161	0.227
Sodium	−0.000	0.997	−0.116	0.354	−0.001	0.995	0.088	0.493
Potassium	0.073	0.596	−0.129	0.351	0.072	0.604	0.186	0.184
Calcium	−0.061	0.653	−0.129	0.336	−0.069	0.609	0.186	0.172
Magnesium	0.021	0.881	−0.086	0.535	0.027	0.845	0.095	0.503
Phosphorus	0.005	0.971	−0.090	0.514	0.009	0.949	0.086	0.541
Iron	0.068	0.600	−0.079	0.543	0.073	0.573	0.080	0.545
Zinc	0.213	0.116	0.081	0.554	0.227	0.096	−0.144	0.299
Copper	0.028	0.838	0.010	0.941	0.052	0.708	−0.083	0.551
Manganese	0.023	0.858	−0.076	0.558	0.042	0.748	0.057	0.667
Retinol	−0.205	0.135	−0.220	0.107	−0.225	0.102	0.287	0.038
Retinol activity equivalent *^3^	−0.123	0.357	−0.232	0.079	−0.139	0.302	0.306	0.022
α-Carotene	0.051	0.694	−0.134	0.293	0.044	0.735	0.203	0.116
β-Carotene	0.023	0.856	−0.123	0.332	0.022	0.862	0.156	0.222
Cryptoxanthin	−0.018	0.895	−0.031	0.820	−0.031	0.818	0.109	0.425
β-Carotene equivalent *^4^	0.023	0.856	−0.123	0.329	0.021	0.869	0.164	0.200
Vitamin D	−0.150	0.248	−0.109	0.399	−0.128	0.326	−0.014	0.916
α-Tocopherol	0.048	0.723	−0.089	0.506	0.034	0.803	0.185	0.172
Vitamin K	0.042	0.759	−0.020	0.885	0.043	0.754	0.073	0.602
Vitamin B_1_	0.213	0.110	−0.058	0.666	0.215	0.107	0.062	0.647
Vitamin B_2_	0.073	0.596	−0.116	0.395	0.058	0.674	0.228	0.099
Dietary nutrient intake								
Niacin	0.093	0.480	−0.093	0.481	0.105	0.429	0.061	0.651
Vitamin B_6_	0.158	0.244	−0.099	0.465	0.169	0.213	0.094	0.496
Vitamin B_12_	−0.135	0.289	−0.094	0.461	−0.119	0.353	−0.002	0.987
Folate	0.024	0.855	−0.112	0.395	0.026	0.846	0.156	0.243
Pantothenic acid	0.125	0.394	−0.039	0.789	0.116	0.432	0.142	0.340
Vitamin C	0.005	0.970	−0.114	0.391	0.006	0.962	0.148	0.271

*^1^ Model were adjusted for maternal age, early-pregnancy BMI at the first clinical visit, gestational weeks, parity, mode of delivery, maternal weight gain, infant sex, and infant birth weight. *^2^ *β* denotes standardized *β* coefficient. *^3^ Sum of retinol, β-carotene/12, α-carotene/24, and cryptoxanthin/24. *^4^ Sum of β-carotene, α-carotene/2, and cryptoxanthin/2. Bold font indicates statistical significance (*p* values < 0.05).

**Table 4:** Multivariable linear regression analysis for human milk macronutrients and infant anthropometric data at 1-month postpartum *^1^.

Exclusively Breast-Fed Infant (n = 29)	Length			Weight		Head Circumference			Chest Circumference			
	*β* *^2^	*p*		*β* *^2^	*p*	*β* *^2^	*p*		*β* *^2^		*p*	
Energy	0.117	0.497		0.197	0.158	0.219	0.223		0.194		0.301	
Protein	−0.088	0.605		−0.158	0.254	−0.109	0.545		−0.187		0.311	
Fat	0.114	0.515		0.187	0.187	0.214	0.239		0.176		0.355	
Carbohydrate	0.126	0.518		0.253	0.105	0.278	0.168		0.386		0.060	
**Exclusively Breast-Fed Infant (n = 29)**	**ΔLength**			**ΔWeight**		**ΔHead Circumference**			**ΔChest Circumference**			
	*β* *^2^	***p***		*β* *^2^	***p***	*β* *^2^	***p***		*β* *^2^		***p***	
Energy	0.025	0.909		0.239	0.247	0.316	0.116		0.220		0.376	
Protein	−0.177	0.408		−0.251	0.241	−0.183	0.364		−0.178		0.467	
Fat	0.010	0.963		0.222	0.288	0.295	0.148		0.191		0.448	
Carbohydrate	0.278	0.255		0.406	0.092	0.284	0.217		**0.540**		**0.044**	
**Exclusively Breast-Fed Infant (n = 29)**	Length SDS *^3^		Weight SDS *^3^		Length SDS *^4^		Weight SDS *^4^		Weight for Length SDS *^4^		Head Circumference SDS *^4^	
	*β* *^2^	***p***	*β* *^2^	***p***	*β* *^2^	***p***	*β* *^2^	***p***	*β* *^2^	***p***	*β* *^2^	***p***
Energy	0.125	0.493	0.207	0.148	0.118	0.493	0.220	0.119	0.134	0.571	0.255	0.205
Protein	−0.090	0.615	−0.152	0.286	0.006	0.973	−0.081	0.571	−0.086	0.711	−0.034	0.867
Fat	0.121	0.513	0.195	0.180	0.114	0.514	0.207	0.149	0.122	0.610	0.249	0.222
Carbohydrate	0.140	0.496	0.309	0.071	0.051	0.796	0.226	0.158	0.224	0.397	0.246	0.281
**Exclusively Breast-Fed Infant (n = 29)**	ΔLength SDS *^3^		ΔWeight SDS *^3^		ΔLength SDS *^4^		ΔWeight SDS *^4^		ΔWeight for Length SDS *^4^		ΔHead Circumference SDS *^4^	
	*β* *^2^	***p***	*β* *^2^	***p***	*β* *^2^	***p***	*β* *^2^	***p***	*β* *^2^	***p***	*β* *^2^	***p***
Energy	0.006	0.977	0.326	0.044	0.047	0.823	0.399	0.046	0.196	0.353	0.323	0.083
Protein	−0.168	0.372	−0.191	0.273	−0.104	0.615	−0.182	0.399	0.015	0.941	−0.120	0.525
Fat	−0.007	0.970	0.318	0.052	0.034	0.873	0.389	0.055	0.198	0.354	0.305	0.108
Carbohydrate	0.251	0.241	0.280	0.155	0.194	0.411	0.280	0.254	0.021	0.930	0.215	0.319

*^1^ Model were adjusted for maternal age, early-pregnancy BMI at the first clinical visit, gestational weeks, parity, mode of delivery, maternal weight gain, infant sex, and birth weight. *^2^ *β* denotes standardized *β* coefficient. *^3^ Calculated based on Japanese growth standard. *^4^ Calculated based on WHO growth standard. Bold font indicates statistical significance (*p* values < 0.05). SDS, standard deviation score.

**Table 5:** Multivariable logistic regression analysis for human milk macronutrients and breast-fed infant growth abnormalities at 1-month postpartum *^1^.

Exclusively Breast-Fed Infant (n = 29)	Length *^2^ (out of 10th %ile–90th %ile)		Weight *^2^ (out of 10th %ile–90th %ile)		Length *^3^ (out of 10th %ile–90th %ile)		Weight *^3^ (out of 10th %ile–90th %ile)	
	OR	95% CI	OR	95% CI	OR	95% CI	OR	95% CI
Energy	1.02	(0.95–1.10)	1.05	(0.94–1.17)	1.00	(0.89–1.11)	1.01	(0.92–1.12)
Protein	2.86	(0.89–9.23)	1.11	(0.37–3.40)	1.08	(0.41–2.82)	1.39	(0.58–3.30)
Fat	1.02	(0.95–1.09)	1.05	(0.95–1.16)	0.94	(0.85–1.05)	1.01	(0.93–1.10)
Carbohydrate	1.12	(0.79–1.60)	0.86	(0.17–4.31)	1.11	(0.72–1.72)	1.65	(0.74–3.68)

*^1^ Model were adjusted for maternal age, early-pregnancy BMI at the first clinical visit, gestational weeks, parity, mode of delivery, maternal weight gain, infant sex, and birth weight. *^2^ Calculated based on Japanese growth standard. *^3^ Calculated based on WHO growth standard. CI, confidence interval; OR, odds ratio.

## Data Availability

The data presented in this study are available on request from the corresponding author.

## Supplementary Materials

The following supporting information can be downloaded at: https://www.mdpi.com/article/10.3390/nu15030654/s1, Supplementary Table S1: Energy-adjusted maternal food intake at 1-month postpartum obtained from brief-type self-administered diet history questionnaire.; Supplementary Table S2: Energy-adjusted maternal nutrient intake at 1-month postpartum obtained from brief-type self-administered diet history questionnaire.; Supplementary Table S3: Multivariable linear regression analysis for maternal food intake and human milk macronutrients at 1-month postpartum.; Supplementary Table S4: Gestational outcome and anthropometric data of exclusively breast-fed and mixed-fed infants.
