# Supplementary material for: Associations between Maternal Diet, Human Milk Macronutrients, and Breast-Fed Infant Growth during the First Month of Life in the SMILE Iwamizawa in Japan
Source: Nutrients. 2023 Jan 28;15(3):654. doi: 10.3390/nu15030654 (PMC9921570; doi:10.3390/nu15030654)
Supplement: Supplementary file 1 [file nutrients-15-00654-s001.zip › nutrients-2134243-supplementary.pdf]

Supplementary Table S1. Energy-adjusted [*1] maternal food intake at 1 month postpartum obtained from brief-type self-administered diet history questionnaire.

Food intake [*2] (n = 71)		Food intake [*2] (n = 71)	
Low fat milk and yogurt (g/day)	41.7 ± 72.4	Citrus fruit (g/day)	18.2 ± 27.5
Milk and yogurt (g/day)	79.9 ± 61.7	Persimmons/strawberries/kiwifruit (g/day)	15.0 ± 21.1
Chicken (g/day)	26.0 ± 16.0	Mayonnaise/dressing (g/day)	5.0 ± 4.3
Pork/beef (g/day)	34.5 ± 22.0	Bread (g/day)	31.6 ± 21.7
Ham/sausage/bacon (g/day)	9.4 ± 6.8	Buckwheat noodles (g/day)	7.8 ± 9.1
Liver (g/day)	0.5 ± 1.3	Japanese wheat noodle (g/day)	17.7 ± 15.1
Squid/octopus/shrimp/shellfish (g/day)	7.5 ± 8.1	Chinese noodles (g/day)	16.0 ± 18.8
Small fish with bones (g/day)	4.5 ± 8.7	Pasta (g/day)	13.5 ± 9.7
Canned tuna (g/day)	3.0 ± 3.4	Green tea (g/day)	50.9 ± 104.8
Dried fish/salted fish (g/day)	10.3 ± 9.1	Black tea/oolong tea (g/day)	52.1 ± 123.2
Oily fish (g/day)	12.1 ± 10.8	Coffee (g/day)	47.6 ± 78.4
Lean fish (g/day)	12.1 ± 9.0	Cola drink/soft drink (g/day)	80.7 ± 145.0
Egg (g/day)	40.8 ± 22.4	100% fruit and vegetable juice (g/day)	56.9 ± 102.8
Tofu/deep-fried tofu (g/day)	30.4 ± 19.3	Sugar (g/day)	0.8 ± 2.0
Natto (g/day)	13.1 ± 12.0	Rice (g/day)	265 ± 102.1
Potatos (g/day)	38.5 ± 26.4	Miso soup (g/day)	126.4 ±
Pickled green leafy vegetables (g/day)	4.4 ± 6.6	Raw fish (g/day)	13.0 ± 14.1
Other pickled vegetables (g/day)	2.8 ± 4.5	Grilled fish (g/day)	24.5 ± 20.0
Lettuces/cabbage (raw) (g/day)	20.7 ± 14.8	Boiled fish (g/day)	57.0 ± 41.7
Green leafy vegetables (g/day)	38.7 ± 31.3	Tempura/fried fish (g/day)	10.8 ± 10.8
Cabbage/Chinese cabbage (g/day)	32.1 ± 18.8	Grilled meat/steak (g/day)	15.0 ± 18.4
Carrots/pumpkin (g/day)	17.5 ± 12.7	Hamburger steak/curry/meat sauce (g/day)	38.2 ± 25.2
Japanese radish/turnip (g/day)	16.3 ± 13.8	Deep-fried foods (g/day)	17.9 ± 15.3
Other root vegetables (g/day)	40.8 ± 25.9	Stir-fried foods (g/day)	59.4 ± 33.1
Tomatoes (g/day)	22.7 ± 23.7	Boiled foods (g/day)	78.7 ± 47.7
Mushrooms (g/day)	9.3 ± 8.4	Soups (g/day)	62.1 ± 37.6
Seaweeds (g/day)	6.1 ± 6.4	Soy sauce (g/day)	1.4 ± 0.3
Western-type confectioneries (g/day)	37.4 ± 29.3	Flavored salt (g/day)	2.8 ± 0.6
Japanese-type confectioneries (g/day)	7.9 ± 10.1	Cooking oil (g/day)	9.8 ± 3.2
Rice crackers/rice cake/okonomiyaki (g/day)	7.2 ± 7.7	Cooking sugar (g/day)	2.8 ± 1.4
Ice cream (g/day)	24.2 ± 26.5		

[*1] Energy adjustment was performed by the residual method. [*2] Means ± SDs.

Supplementary Table S2. Energy-adjusted [*1] maternal nutrient intake at 1 month postpartum obtained from brief-type self-administered diet history questionnaire.

Nutrient intake [*2] (n = 71)	
Energy (kcal/day)	1594.5 ± 448.7
Protein (g/day)	58.4 ± 8.3
Animal protein (g/day)	33.8 ± 8.4
Vegetable protein (g/day)	24.6 ± 3.6
Fat (g/day)	50.5 ± 7.7
Animal fat (g/day)	24.0 ± 5.9
Vegetable fat (g/day)	26.4 ± 5.3
Saturated fat (g/day)	14.7 ± 3.0
Monounsaturated fat (g/day)	18.1 ± 3.2
Polyunsaturated fat (g/day)	11.3 ± 1.7
n-3 Polyunsaturated fat (g/day)	2.1 ± 0.5
n-6 Polyunsaturated fat (g/day)	9.1 ± 1.4
Cholesterol (mg/day)	362.7 ± 107.0
Carbohydrate (g/day)	220.3 ± 24.2
Sucrose (g/day)	13.3 ± 8.2
Total dietary fiber (g/day)	10.0 ± 3.0
Soluble dietary fiber (g/day)	2.6 ± 0.8
Insoluble dietary fiber (g/day)	7.2 ± 2.1
Minerals (g/day)	14.6 ± 2.3
Sodium (mg/day)	3356.3 ± 539.1
Potassium (mg/day)	2041.9 ± 519.9
Calcium (mg/day)	460.7 ± 138.9
Magnesium (mg/day)	191.2 ± 40.4

Nutrient intake [*2] (n = 71)	
Phosphorus (mg/day)	869.7 ± 145.1
Iron (mg/day)	6.3 ± 1.5
Zinc (mg/day)	7.1 ± 0.8
Copper (mg/day)	0.9 ± 0.2
Manganese (mg/day)	2.1 ± 0.5
Retinol (μg/day)	306.0 ± 188.3
Retinol activity equivalents [*3] (μg/day)	336.2 ± 236.1
α-Carotene (μg/day)	2852.2 ± 1719.0
β-Carotene (μg/day)	270.7 ± 277.6
Cryptoxanthin (μg/day)	3159.6 ± 1870.9
β-Carotene equivalents [*4] (μg/day)	572.6 ± 259.6
Vitamin D (μg/day)	9.2 ± 5.0
α-Tocopherol (mg/day)	6.4 ± 1.4
Vitamin K (μg/day)	262.7 ± 126.4
Vitamin B_1 (mg/day)	0.7 ± 0.1
Vitamin B_2 (mg/day)	1.1 ± 0.3
Niacin (mg/day)	12.2 ± 2.7
Vitamin B_6 (mg/day)	1.0 ± 0.2
Vitamin B_{12}, μg/day	6.3 ± 2.7
Folate (μg/day)	256.8 ± 94.6
Pantothenic acid (mg/day)	5.7 ± 1.0
Vitamin C (mg/day)	90.9 ± 44.6

[*1] Energy adjustment was performed by the residual method. [*2] Means ± SDs. [*3] Sum of retinol, β-carotene/12, α-carotene/24, and cryptoxanthin/24. [*4] Sum of β-carotene, α-carotene/2, and cryptoxanthin/2.

Supplementary Table S3. Multivariable linear regression analysis for maternal food intake and human milk macronutrients at 1 month postpartum [*1].

(n = 71)	Human milk macronutrient							
	Energy		Protein		Fat		Carbohydrate	
	β [*2]	*P*	β [*2]	*P*	β [*2]	*P*	β [*2]	*P*
Food intake								
Low fat milk and yogurt	0.106	0.438	-0.007	0.961	0.084	0.539	0.128	0.350
Milk and yogurt	-0.186	0.174	-0.094	0.493	-0.181	0.186	0.065	0.644
Chicken	0.144	0.267	0.075	0.561	0.137	0.292	0.007	0.957
Pork/beef	0.201	0.110	0.069	0.588	0.206	0.103	-0.112	0.384
Ham/sausage/bacon	0.070	0.584	-0.020	0.874	0.061	0.634	0.055	0.671
Liver	-0.237	0.098	-0.196	0.170	-0.256	0.074	0.267	0.065
Squid/octopus/shrimp/shellfish	-0.105	0.424	0.125	0.337	-0.100	0.447	-0.121	0.366
Small fish with bones	-0.223	0.081	-0.041	0.748	-0.216	0.092	-0.012	0.926
Canned tuna	-0.043	0.734	-0.096	0.444	-0.042	0.738	0.085	0.504
Dried fish/salted fish	-0.128	0.320	-0.005	0.966	-0.115	0.373	-0.056	0.669
Oily fish	0.026	0.847	-0.208	0.114	0.048	0.718	0.045	0.737
Lean fish	0.067	0.606	-0.084	0.513	0.092	0.479	-0.006	0.962
Egg	0.099	0.457	0.020	0.878	0.076	0.570	0.086	0.526
Tofu/deep-fried tofu	0.102	0.457	-0.011	0.936	0.131	0.341	-0.148	0.287
Natto	-0.004	0.981	0.075	0.612	-0.002	0.987	-0.016	0.914
Potatos	0.049	0.732	-0.070	0.618	0.055	0.702	0.093	0.518
Pickled green leafy vegetables	0.017	0.899	-0.010	0.938	-0.003	0.981	0.127	0.347
Other pickled vegetables	0.075	0.577	0.059	0.658	0.084	0.532	-0.119	0.381
Lettuces/cabbage (raw)	0.207	0.109	0.186	0.148	0.213	0.100	-0.137	0.298
Green leafy vegetables	0.002	0.988	-0.081	0.512	0.009	0.943	0.064	0.608
Cabbage/Chinese cabbage	0.133	0.304	-0.144	0.266	0.134	0.302	0.181	0.168
Carrots/pumpkin	0.052	0.688	-0.133	0.300	0.045	0.727	0.201	0.122
Japanese radish/turnip	0.174	0.212	-0.056	0.689	0.167	0.233	0.098	0.493
Other root vegetables	0.238	0.056	-0.071	0.571	0.242	0.052	0.116	0.365
Tomatoes	-0.068	0.606	-0.116	0.374	-0.068	0.605	0.108	0.416
Mushrooms	0.166	0.215	-0.039	0.769	0.192	0.151	-0.058	0.673
Seaweeds	-0.065	0.610	-0.033	0.797	-0.054	0.674	-0.024	0.853
Western-type confectioneries	0.110	0.413	0.034	0.801	0.126	0.348	-0.096	0.483
Japanese-type confectioneries	-0.100	0.465	0.095	0.488	-0.103	0.453	-0.097	0.484
Rice crackers/rice cake/okonomiyaki	0.119	0.361	0.121	0.348	0.123	0.344	-0.146	0.268
Ice cream	-0.022	0.875	-0.092	0.497	-0.045	0.742	0.183	0.184
Citrus fruit	0.023	0.865	0.029	0.826	0.014	0.919	0.054	0.694
Persimmons/strawberries/kiwifruit	-0.193	0.140	-0.081	0.538	-0.179	0.173	0.009	0.946
Mayonnaise/dressing	0.209	0.126	0.176	0.197	0.169	0.217	0.066	0.636

[*1] Model were adjusted for maternal age, early-pregnancy BMI at the first clinical visit, gestational weeks, parity, mode of delivery, maternal weight gain, infant sex, and birth weight. [*2] β denotes standardized β coefficient. Bold font indicates statistical significance (*P* values < 0.05).

Supplementary Table S3, continued [*1].

(n = 71)	Human milk macronutrient							
	Energy		Protein		Fat		Carbohydrate	
	β [*2]	*P*	β [*2]	*P*	β [*2]	*P*	β [*2]	*P*
Food intake								
Bread	-0.113	0.383	-0.008	0.949	-0.153	0.238	0.217	0.096
Buckwheat noodles	0.007	0.957	-0.017	0.893	0.015	0.909	0.004	0.977
Japanese wheat noodle	-0.047	0.715	0.120	0.349	-0.057	0.659	-0.054	0.678
Chinese noodles	-0.059	0.654	-0.063	0.630	-0.065	0.625	0.068	0.612
Pasta	0.152	0.232	-0.026	0.835	0.142	0.266	0.019	0.886
Green tea	0.102	0.426	-0.044	0.730	0.108	0.405	0.087	0.506
Black tea/oolong tea	-0.123	0.350	-0.159	0.227	-0.131	0.319	0.238	0.072
Coffee	-0.020	0.878	-0.043	0.744	-0.042	0.753	0.135	0.314
Cola drink/soft drink	-0.247	0.067	-0.189	0.161	-0.253	0.061	0.224	0.102
100% fruit and vegetable juice	-0.040	0.770	-0.060	0.659	-0.051	0.709	0.095	0.495
Sugar	-0.219	0.077	-0.089	0.474	-0.214	0.085	0.086	0.500
Rice	-0.016	0.900	0.086	0.509	0.008	0.951	-0.226	0.085
Miso soup	-0.036	0.786	-0.138	0.290	-0.014	0.916	-0.008	0.953
Raw fish	**-0.287**	**0.028**	-0.059	0.655	**-0.280**	**0.032**	0.028	0.834
Grilled fish	-0.112	0.371	-0.172	0.168	-0.118	0.350	0.164	0.196
Boiled fish	0.168	0.191	0.021	0.873	0.183	0.155	-0.075	0.569
Tempura/fried fish	-0.254	0.064	-0.076	0.585	-0.233	0.092	-0.017	0.904
Grilled meat/steak	0.053	0.680	0.046	0.721	0.046	0.723	0.008	0.950
Hamburger steak/curry/meat sauce	0.020	0.876	-0.116	0.352	0.036	0.777	-0.024	0.853
Deep-fried foods	0.095	0.483	0.170	0.205	0.072	0.598	-0.068	0.623
Stir-fried foods	0.084	0.530	0.067	0.617	0.065	0.629	0.044	0.746
Boiled foods	0.131	0.306	-0.109	0.393	0.148	0.248	0.030	0.820
Soups	-0.032	0.803	0.013	0.919	-0.039	0.758	0.017	0.894
Soy sauce	0.115	0.385	-0.028	0.833	0.106	0.424	0.037	0.784
Flavored salt	0.119	0.341	-0.137	0.274	0.133	0.289	0.059	0.645
Cooking oil	0.053	0.700	0.069	0.619	0.039	0.781	-0.010	0.941
Cooking sugar	0.187	0.144	-0.067	0.604	0.208	0.106	-0.019	0.887

[*1] Model were adjusted for maternal age, early-pregnancy BMI at the first clinical visit, gestational weeks, parity, mode of delivery, maternal weight gain, infant sex, and birth weight. [*2] β denotes standardized β coefficient. Bold font indicates statistical significance (*P* values < 0.05).

Supplementary Table S4. Gestational outcome and anthropometric data of exclusively breast-fed and mixed-fed infants.

	Exclusively breast-fed infant (n = 29)	Mixed-fed infant (n = 42)	*P*
Gestational outcome			
Male [n (%)]	18 (62.1)	23 (54.8)	0.539
Gestational period (weeks) *1	39.3 ± 1.2	39.6 ± 1.2	0.274
Primipara [n (%)]	9 (31.0)	19 (45.2)	0.226
Vaginal delivery [n (%)]	25 (86.2)	36 (85.7)	0.953
Birth weight (g) *1	3211.9 ± 289.1	3258.8 ± 451.5	0.596
Birth weight for gestational age *2			
SGA [n (%)]	0 (0.0)	3 (7.1)	0.072
LGA [n (%)]	7 (24.1)	11 (26.2)	0.845
Low birth weight delivery [< 2,500g; n (%)]	0 (0.0)	2 (4.8)	0.144
Preterm delivery [< 37weeks; n (%)]	1 (3.4)	0 (0.0)	0.178
Anthropometric data at 1 month of age *1			
Length (cm)	55.0 ± 1.6	54.7 ± 2.2	0.554
Weight (g)	4441.4 ± 472.4	4442.3 ± 615.9	0.995
Head circumference (cm)	37.1 ± 1.1	37.3 ± 1.1	0.604
Chest circumference (cm)	36.3 ± 1.6	36.2 ± 1.9	0.854
Length SDS *3	0.88 ± 0.72	0.77 ± 1.00	0.630
Weight SDS *3	0.24 ± 0.75	0.25 ± 0.98	0.947
Length SDS *4	0.57 ± 0.81	0.28 ± 0.99	0.195
Weight SDS *4	0.25 ± 0.73	0.11 ± 0.90	0.487
Weight for length SDS *4	-0.35 ± 0.86	-0.19 ± 1.28	0.522
Head circumference SDS *4	0.34 ± 0.77	0.35 ± 0.78	0.978
Changes in anthropometric data during 1 month of age *1			
ΔLength (cm)	5.9 ± 1.6	5.2 ± 1.8	0.057
ΔWeight (g)	1229.5 ± 299.4	1183.5 ± 411.8	0.609
ΔHead circumference (cm)	3.5 ± 1.4	3.4 ± 1.4	0.791
ΔChest circumference (cm)	4.0 ± 1.5	3.8 ± 1.7	0.647
ΔLength SDS *3	0.77 ± 0.90	0.49 ± 0.96	0.214
ΔWeight SDS *3	-0.29 ± 0.69	-0.34 ± 0.93	0.781
ΔLength SDS *4	0.41 ± 0.78	0.01 ± 0.90	0.058
ΔWeight SDS *4	0.03 ± 0.50	-0.02 ± 0.67	0.757
ΔWeight for length SDS *4	-0.48 ± 1.11	-0.10 ± 1.35	0.213
ΔHead circumference SDS *4	0.44 ± 1.16	0.40 ± 1.10	0.900

*1 Means ± SDs. *2 SGA and LGA were defined as the birth weight < 10th percentile and ≥ 90th percentile for gestational age, respectively. *3 Calculated based on Japanese growth standard. *4 Calculated based on WHO growth standard. LGA, large for gestational age; SDS, standard deviation score; SGA, small for gestational age.
